# Ultrathin and Stable Aromatic Monolayers on Naturally Oxidized Aluminum: The Role of Type and Number of Anchoring Groups

**DOI:** 10.1021/acs.jpclett.6c02547

**Published:** 2026-09-16

**Authors:** Anna Rojek, Marta Szatny, Daria M. Cegiełka, Łukasz Bodek, Yoshiaki Shoji, Takanori Fukushima, Michael Zharnikov, Piotr Cyganik

**Affiliations:** § Jagiellonian University, Faculty of Physics, Astronomy and Applied Computer Science, Smoluchowski Institute of Physics, Łojasiewicza 11, 30-348 Kraków, Poland; # Laboratory for Chemistry and Life Science, Institute of Integrated Research, Institute of Science Tokyo, Yokohama 226-8501, Japan; ‡ Research Center for Autonomous Systems Materialogy (ASMat), Institute of Integrated Research, Institute of Science Tokyo, Yokohama 226-8501, Japan; † Institute for Physical Chemistry, Heidelberg University, Im Neuenheimer Feld 253, D-69120 Heidelberg, Germany

## Abstract

Ultrathin aromatic self-assembled monolayers (SAMs) on aluminum oxide are attractive interfacial layers for organic electronics, yet their stability remains a key challenge. Here, we investigate the formation, structure, thermal stability, and chemical robustness of phenyl- and triptycene-based SAMs deposited on naturally oxidized aluminum (∼1 nm AlOx). The effects of anchoring group chemistry (carboxylic vs phosphonic acid) and bonding mode (monopodal vs tripodal) are systematically compared. X-ray photoelectron spectroscopy (XPS) and near-edge X-ray absorption fine structure spectroscopy confirm the formation of densely packed, upright-oriented ∼1 nm thick monolayers irrespective of molecular architecture. Thermally programmed XPS reveals that phosphonic acid anchoring significantly increases desorption energies relative to carboxylic acid analogues, while tripodal binding provides an additional stability enhancement, yielding a high desorption energy of ∼1.9 eV. Exchange experiments demonstrate exceptional chemical robustness of tripodal phosphonic acid monolayers. These findings establish phosphonic acid anchoring combined with tripodal binding as an effective strategy for engineering ultrathin, highly stable aromatic interfaces on technologically relevant oxide surfaces.

## Graphical abstract

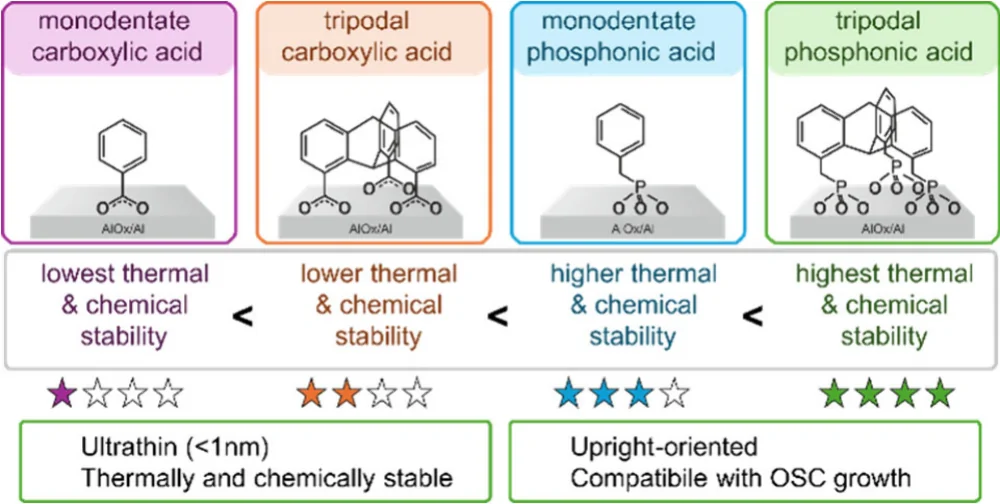

Surface engineering of aluminum oxide (AlO_*x*_) with organic films is pivotal for the fabrication of organic field-effect transistors (OFETs),^1–4^ organic light-emitting devices (OLEDs),^5–7^ and solar cells.^5,8^ Such engineering is most often achieved using self-assembled monolayers (SAMs),^9,10^ which, in the case of OFETs, enable control over the interface between the organic semiconductor (OSC) and the aluminum-oxide-based gate dielectric.^5,11,12^ Considering that the conduction channel in OFETs is located at this interface and is only a few nanometers thick, improving the OSC morphology and suppressing charge trapping at the interface through SAM engineering of the aluminum oxide electrode enables control over the *I*–*V* characteristics, leading to more than an order of magnitude improvement in the key parameters such as charge mobility, on/off ratio, and threshold voltage.^1^ Generally, for OFET-based applications, SAMs formed on the AlO_*x*_ surface should exhibit four key features, i.e., (1) they should contain an upright-oriented aromatic structure to promote well-defined growth of an OSC typically based on aromatic molecules such as pentacene,^13^ which provides the highest known charge mobility;^14^ (2) they should be as thin as possible to reduce the junction capacitance and thus increase the source–drain current at low operating voltage;^11^ (3) they should exhibit high thermal and chemical stability to withstand both the device fabrication process and heat dissipation during its operation; and (4) they should allow modification of their molecular structure to control the morphology of the deposited OSC and the interfacial dipole. In our recent studies,^15,16^ we analyzed the formation of long-chain aliphatic SAMs on naturally oxidized aluminum, where the naturally formed AlO_*x*_ overlayer, typically ∼1 nm thick, may serve as a simple and inexpensive ultrathin dielectric layer. The detailed analysis of this model system demonstrated that the structural quality and stability of SAMs on this substrate depend on the selection of the anchoring group, such as carboxylic or phosphonic acids (abbreviated hereafter as CA and PA, respectively), with the latter group yielding much better performance. However, PA-anchored, long-chain aliphatic SAMs are hardly relevant for the gate electrode engineering in OFETs, because of their thickness, molecular structure, and inferior electrical conductivity. Aromatic monolayers will suit this purpose better. Therefore, in the current study, we analyze for the first time the thinnest aromatic SAM, i.e., with a thickness corresponding to a single phenyl ring, formed on naturally oxidized aluminum. To optimize the structure and stability of such an ultrathin aromatic monolayer, we used either CA or PA anchoring groups and, importantly, two different bonding modes, i.e., either monopodal, based on the phenyl backbone, or tripodal, based on a triptycene framework. The latter moiety represents a rigid propeller-shaped structure consisting of three phenyl rings, which, if decorated with the CA or PA groups, become potentially capable of anchoring to the AlO_*x*_ substrate in a tripodal fashion (see Figure 1a–d for schematic presentation). This specific aspect of system optimization, which exploits the idea of multipodal binding, is inspired by the *chelate effect*,^17^ where multipodal binding groups provide higher thermodynamic stability due to the disorder entropy change during bonding, which is lower for multipodal ligands. The *chelate effect*, however, is usually considered for processes in solution in contrast to on-substrate chemisorption in our case, where many additional enthalpic and entropic contributions come into play, such as those related to the formation of a 2D structure by ligands, molecule–substrate bonding reconfiguration, and possible relaxation/reconstruction of the substrate surface. Owing to these additional factors, the stability enhancement associated with multipodal binding is difficult to predict for a given SAM system and must therefore be evaluated experimentally. In this context, our recent study of CA-anchored SAMs on a silver substrate, which revealed the markedly enhanced thermal and chemical stability of triptycene-based films relative to their monodentate counterparts, provided direct inspiration for the present investigation of multipodal binding.^18^ The present study extends and generalizes this previous work by addressing the technologically relevant AlO_*x*_ substrate, which differs fundamentally from silver, and by investigating not only CA-anchored but also PA-anchored SAMs. The selected substrate not only is of relevance and importance on its own but also represents a testbed case for other oxide materials, broadly used in electronics and photovoltaics. Tripodal triptycene-based molecules studied here can be flexibly functionalized with different tail groups,^19^ allowing their adaptation to specific purposes, such as control of interfacial dipole or surface wetting properties, which contribute to device performance.

**Figure 1:**
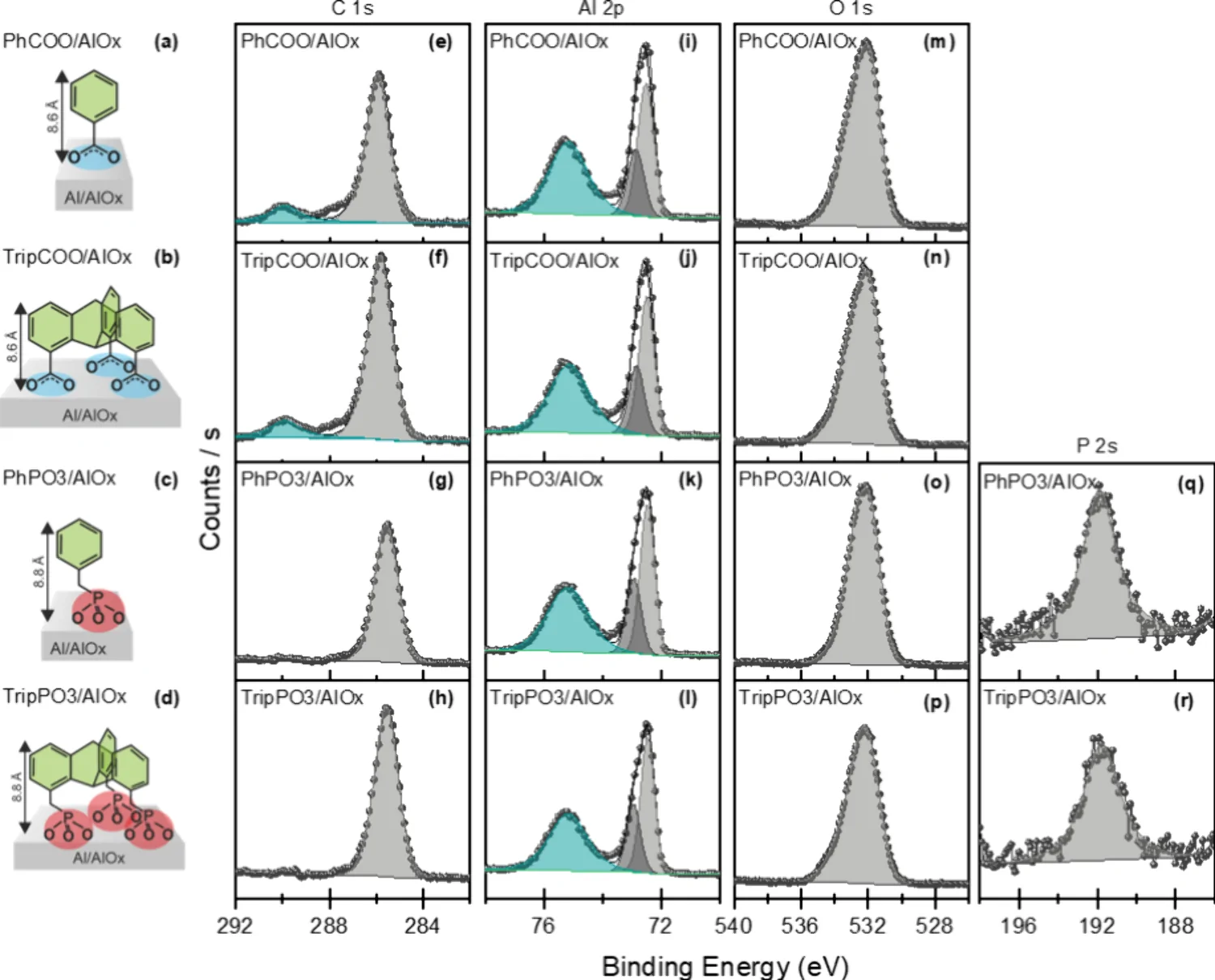
Schematics of the PhCOO, TripCOO, PhPO_3_, and TripPO_3_ molecules adsorbed on Al/AlO_*x*_ substrates (a–d), together with the representative XPS data (e–r) for the respective monolayers. In the consecutive columns from left to right, the C 1s (e–h), Al 2p (i–l), O 1s (m–p), and P 2s (q, r) spectra are presented.

Representative XPS data obtained for PhCOO­(PO_3_)/AlO_*x*_ and TripCOO­(PO_3_)/AlO_*x*_ are presented in Figure 1. For PhCOO/AlO_*x*_, the C 1s spectrum is represented by two peaks at binding energies (BEs) of ∼285.9 and ∼289.8 eV, assigned to the phenyl backbone and CA group, respectively (Figure 1e). The above BE values are noticeably higher than those for the analogous monolayers on Ag(111) (∼283.9 and ∼287.0 eV, respectively),^18,20–22^ corresponding to upward shifts of the respective peaks by ∼2.0 and ∼2.8 eV, respectively, and indicating a strong electrostatic effect associated with the formation of an interfacial dipole layer.^23–27^ According to the previous analysis of long-chain aliphatic SAMs on the analogous substrates (CH_3_-(CH_2_)_15_-COO/AlO_*x*_, HDCOO/AlO_*x*_), this effect originates from the inherent dipole layer formed by the thin oxide layer, which shifts backbone and carboxylate C 1s components by ∼1.3 and ∼3.0 eV, respectively.^15,16,28^ The larger shift observed here (∼2.0 eV) for the main phenyl component can be associated with the reduced distance from the substrate of the C atoms in a single phenyl ring compared to the average distance of C atoms in a long aliphatic chain of HDCOO/AlO_*x*_. Consistently, the magnitude of this shift (∼3.0 eV), observed for the carboxylic component, is the same for PhCOO/AlO_*x*_ and HDCOO/AlO_*x*_, as in both monolayers the respective C atoms are located at similar distances from the AlO_*x*_ substrate. Such an explanation of the observed shifts in BE for the C 1s components is also confirmed by the data obtained for TripCOO/AlO_*x*_ (Figure 1f), for which the peaks assigned to the CA group and triptycene (at ∼289.8 eV and ∼285.8 eV, respectively) are both located at BEs similar to those for PhCOO/AlO_*x*_. The close resemblance of the CA-related features in the spectra of PhCOO/AlO_*x*_ and TripCOO/AlO_*x*_ suggests similar adsorption configurations for all three CA anchoring groups of TripCOO in the latter SAM, which corresponds to the expected tripodal bonding mode. As expected, the C 1s spectra of PhPO_3_/AlO_*x*_ and TripPO_3_/AlO_*x*_ exhibit only one peak, corresponding to the molecular backbone. The BE of this peak, ∼285.5 eV, is lower by ∼0.4 eV than that in PhCOO/AlO_*x*_ and TripCOO/AlO_*x*_ (∼285.9 eV). This observation is fully consistent with our former analysis for the aliphatic monolayers with the same anchoring groups, i.e., HDCOO/AlO_*x*_ and HDPO_3_/AlO_*x*_ (CH_3_-(CH_2_)_15_-PO_3_/AlO_*x*_).^16^ The BE position of the C 1s peak for the latter system was consistently lower (depending on the solvent used for SAM formation) than that for the former one, with a difference of ∼0.4 eV for isopropyl alcohol. This behavior was explained by the presence of an additional dipole layer associated with the PA anchoring group and oriented in the opposite direction compared to the dipole layer stemming from the AlO_*x*_ substrate.^16^ We believe that the same situation occurs for both PhPO_3_/AlO_*x*_ and TripPO_3_/AlO_*x*_. The C 1s spectra were also used to estimate the SAM thickness using hexadecanethiol (HDT) on the Au(111) substrate as a reference system with known thickness (see the Supporting Information for details of these calculations). These estimates, summarized in Table 1, show that the effective thicknesses for all analyzed SAMs are similar and within the range of ca. 0.9–1.1 nm, which is close to the geometrical thickness of these monolayers (∼0.9 nm) for the fully upright oriented molecules. Thus, the thickness analysis confirms the formation of well-defined, densely packed SAMs for all analyzed systems. The Al 2p spectra shown in Figures 1i–l exhibit two main components related to the metallic aluminum (∼72.8 eV) and aluminum oxide (∼75.2 eV). Taking the intensity ratio of these two components and considering signal attenuation, the effective thickness of the oxide layer was estimated (see the Supporting Information for details). For all analyzed monolayers, a similar value of ∼0.9–1.0 nm was obtained, which is fully consistent with our previous analyses^15,16^ for this type of substrate. As a next step, the O/Al ratio was estimated based on the intensity ratio of the O 1s (Figures 1m–p) and Al 2p (oxide component) signals (see the Supporting Information for details). The value of ∼1.60 obtained for all analyzed samples is also fully consistent with our former analyses^15,16^ and indicates that the stoichiometry of the thin oxide layer is close to Al_2_O_3_.

**Table 1:** Overview of the Parameters (Average Values) for the Analyzed SAMs on AlO_*x*_

Parameter	PhCOO/AlO_*x*_	TripCOO/AlO_*x*_	HDCOO/AlO_*x*_	PhPO_3_/AlO_*x*_	TripPO_3_/AlO_*x*_	HDPO_3_ /AlO_*x*_
Effective thickness (nm)	1.02 ± 0.05	1.15 ± 0.05	1.87 ± 0.10	0.90 ± 0.05	0.95 ± 0.05	2.03 ± 0.10
Tilt angle (degrees)	35 ± 3	34 ± 3	36 (ref^15^.)	36 ± 3	38 ± 3	31 (ref^16^.)
Desorption temperature, *T*_D_ (K)	505 ± 30	556 ± 15^a^	364 ± 12^a^	573 ± 3	603 ± 21	585 ± 10
Desorption Energy, *E*_D_ (eV)	1.53 ± 0.09	1.68 ± 0.04^a^	1.10 ± 0.03^a^	1.75 ± 0.02	1.86 ± 0.07	1.79 ± 0.03

a For TripCOO/AlO_*x*_ and HDCOO/AlO_*x*_, the *T*_D_ and *E*_D_ values listed in the table refer to the first desorption event, which represents the loss of the original SAM structure. The second desorption feature is attributed to a thermally induced phase formed after substantial desorption of the original monolayer.

Note that the O 1s spectra of all the studied SAMs exhibit only one peak, originating primarily from the AlO_*x*_ layer, with the oxygen atoms of the SAM anchoring groups providing minor contributions only. Finally, for PhPO_3_/AlO_*x*_ and TripPO_3_/AlO_*x*_, the phosphorus signal was analyzed using the P 2s spectra (Figure 1q,r), taking into account that the P 2p features, which are usually used for analysis of PA-anchored SAMs,^29–31^ overlap in the present case with the plasmon loss satellite of the Al 2s peaks.^32^ For both monolayers, the BE of this peak, ∼191.9 eV, is consistent with the value expected for the anchoring −PO_3_ group; however, the exact character of the bonding configuration, which may adopt monodentate, bidentate, or tridentate form,^33,34^ cannot be identified from the P 2s signal (and from the P 2p spectra as well), which is not sufficiently sensitive to such differences.^29^ We note, however, that the intensity of the P 2s signal for PhPO_3_/AlO_*x*_ and TripPO_3_/AlO_*x*_ is very similar (within ±5%), indicating that the averaged area per TripPO_3_ molecule on the AlO_*x*_ substrate is practically equivalent to the average area occupied by three PhPO_3_ molecules. Considering the similar film thicknesses measured for all studied SAMs, we may suppose that the above molecular footprint relation is also true for PhCOO/AlO_*x*_ and TripCOO/AlO_*x*_.

In the following step, the structure of all monolayers was analyzed by NEXAFS spectroscopy, with the corresponding C K-edge data presented in Figures 2a–d. For electronic structure analysis, the spectra were collected at the so-called “magic” incidence angle of the X-ray beam (∼55°), as they are not affected by the molecular orientation.^35^ The most pronounced resonance is associated with the phenyl rings (*π*_ph_^*^) at a photon energy (PE) of ∼284.9 and ∼285.2 eV for the monolayers based on a single phenyl ring^34^ (PhCOO/AlO_*x*_ or PhPO_3_/AlO_*x*_) and triptycene moiety (TripCOO/AlO_*x*_ or TripPO_3_/AlO_*x*_), respectively (Figures 2a–d).^18,30^ The second resonance for PhCOO/AlO_*x*_ and TripCOO/AlO_*x*_ at a PE of ∼288.5 eV, visible in Figures 2a,b, is associated with the carboxylate anchoring group (*π*_coo_^*^) and was also reported for the analogous SAMs on silver.^18,36^ For PhPO_3_/AlO_*x*_ and TripPO_3_/AlO_*x*_ (Figures 2c,d), a similar but noticeably less intense resonance at ∼288.7 eV is recorded. This feature can be tentatively assigned to residual contamination (C=O and COOH) which, obviously, could not be completely removed upon the molecular assembly (*π*_ct_^*^).^30^ For all analyzed samples, a pronounced linear dichroism was observed, as shown by the difference spectra taken between normal (90°) and grazing (20°) incidence of the X-ray beam, which exhibit a positive peak at the position of the *π*_ph_^*^ resonance, thus confirming upright orientation of the related phenyl rings (with respect to the substrate plane). Following the established procedure,^20,35–37^ the average tilt angle of the molecular axis (*β*) for the studied systems (see the schematic cartoons in Figures 2i–l) was estimated by analyzing the dependence of the *π*_ph_^*^ resonance intensity on the X-ray beam incidence angle within the theoretical framework^35^ for a vector-type orbital (see the Supporting Information for more details). The results of these calculations are summarized in Table 1 and show for all four monolayers similar tilt angles in the range of 34–38°, which are by ∼10° larger compared to the analogous parameters for PhCOO/Ag and TripCOO/Ag (24 ± 3°)^18^ but similar to the values for the PhPO_3_ and TripPO_3_ SAMs on indium tin oxide (29 ± 3°).^30^ We note at this point that the comparably large tilt angle (24 ± 3°) reported earlier for TripCOO/Ag was not related to the non-tripodal anchoring of the molecules to the Ag(111) substrate, which was verified by the respective XPS analysis, but most probably resulted from bonding some of the anchoring groups (one or two) of triptycene to the Ag adatoms with remaining groups bonded to the *in-plane* surface.^18^ For the systems analyzed here, the information on bonding configuration is not accessible; therefore, we can only speculate that the naturally formed oxide film is certainly much rougher on the atomic scale compared to the Ag(111) surface and, therefore, may result in even more tilted adsorption configurations of the triptycene framework. Considering significant tilting of phenyl rings in aromatic monolayers analyzed here, one could expect respective film thicknesses lower than those measured here by XPS, which are in fact close to the fully upright oriented configuration. This discrepancy is most likely caused by adventitious carbon contamination, which is difficult to completely avoid given the slightly polar character of the aromatic rings. This interpretation is further supported by the effective thicknesses measured for the strongly hydrophobic reference monolayers formed by HDCOO/AlO_*x*_ and HDPO_3_/AlO_*x*_. In these cases, the measured effective thicknesses are fully consistent with the corresponding geometrical thicknesses corrected for the molecular tilt angle determined by NEXAFS spectroscopy. For example, for HDPO_3_/AlO_*x*_, assuming a geometrical thickness of approximately 2.35 nm for fully upright molecules and a tilt angle of 31° of the aliphatic chain, an effective film thickness of approximately 2.04 nm is expected, in excellent agreement with the experimentally determined value.

**Figure 2:**
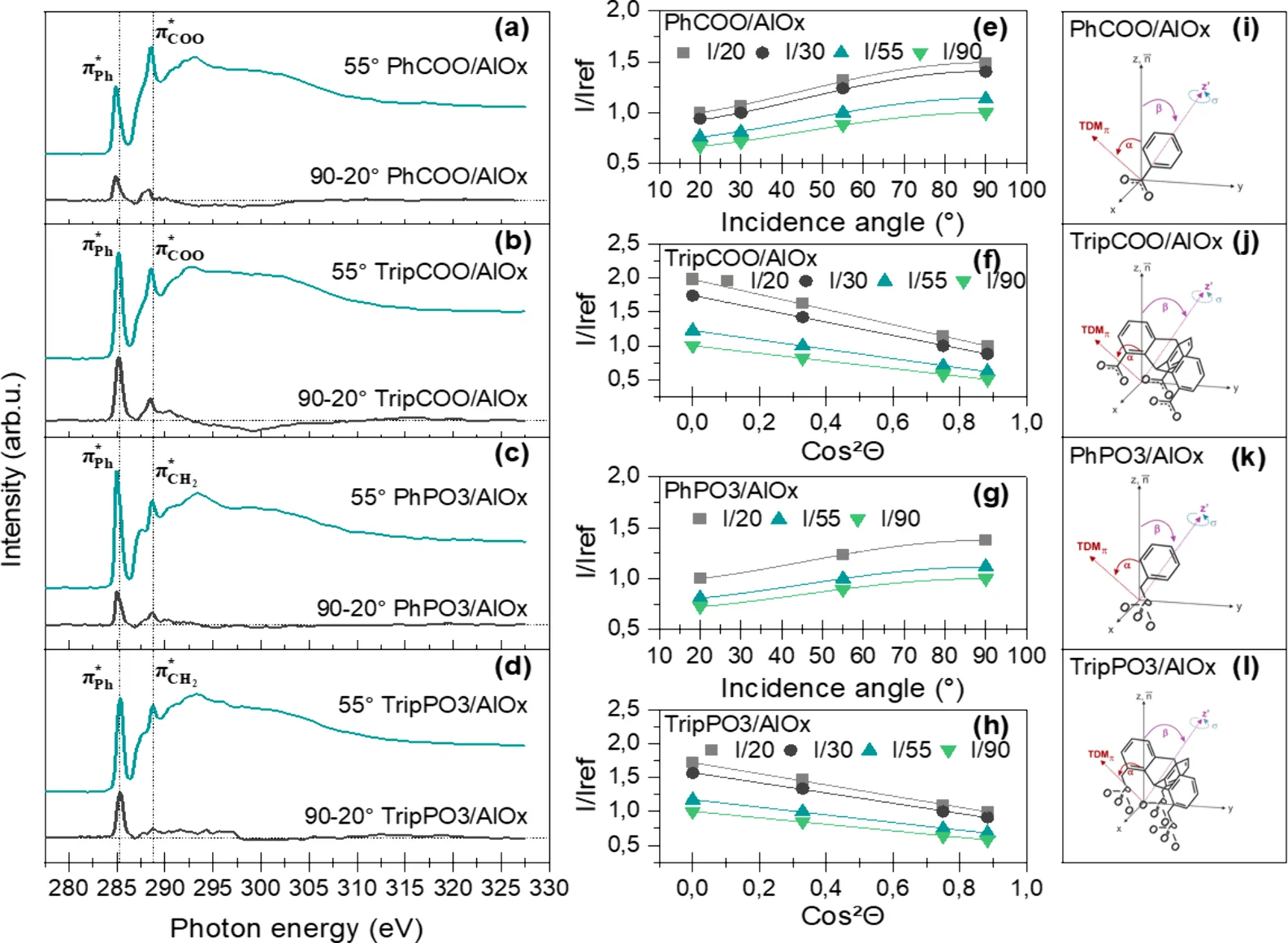
(a–d) C K-edge NEXAFS spectra of PhCOO/AlO_*x*_ (a), TripCOO/AlO_*x*_ (b), PhPO_3_/AlO_*x*_ (c), and TripPO_3_/AlO_*x*_ (d) taken at an X-ray incident angle of 55° (green lines) with indicated most prominent resonances (*π*_ph_^*^ and *π*_coo_^*^) and the respective difference spectra (black lines) between normal (90°) and grazing (20°) incidence. (e–h) The analysis of the tilt (β) of the molecules relative to the substrate normal for these monolayers based on the intensity of the *π*_ph_^*^ resonance obtained at different X-ray incidence angles of 20°, 30°, 55°, and 90°. (i–l) Schematic presentation of the calculated tilt angles together with the TDM tilt angle (α) and the twist angle (σ). See text and the Supporting Information for details.

The thermal stability of PhCOO­(PO_3_)/AlO_*x*_ and TripCOO­(PO_3_)/AlO_*x*_ was quantitatively assessed using thermally programmed XPS (TP-XPS). Within the applied procedure, the intensities of the C 1s and Al 2p signals were monitored while the sample temperature was continuously increased from room temperature to 800 K at a rate of 3.6 K/min. The respective spectra were recorded at 5 K intervals, with each spectrum taken at a different spot on the sample (selected from the predefined matrix; see the Supporting Information for details) to avoid possible irradiation-induced damage.^38^ For PhCOO/AlO_*x*_, a progressive decrease of the main C 1s signal at ∼285.9 eV, associated with the aromatic backbone, was observed with increasing sample temperature (Figure 3a). The corresponding desorption temperature was determined from the maximum of a Gaussian function fitted to the derivative of this signal (Figure 3b). The average value of the desorption temperature obtained from several (see above) independent measurements is 505 ± 30 K.

**Figure 3:**
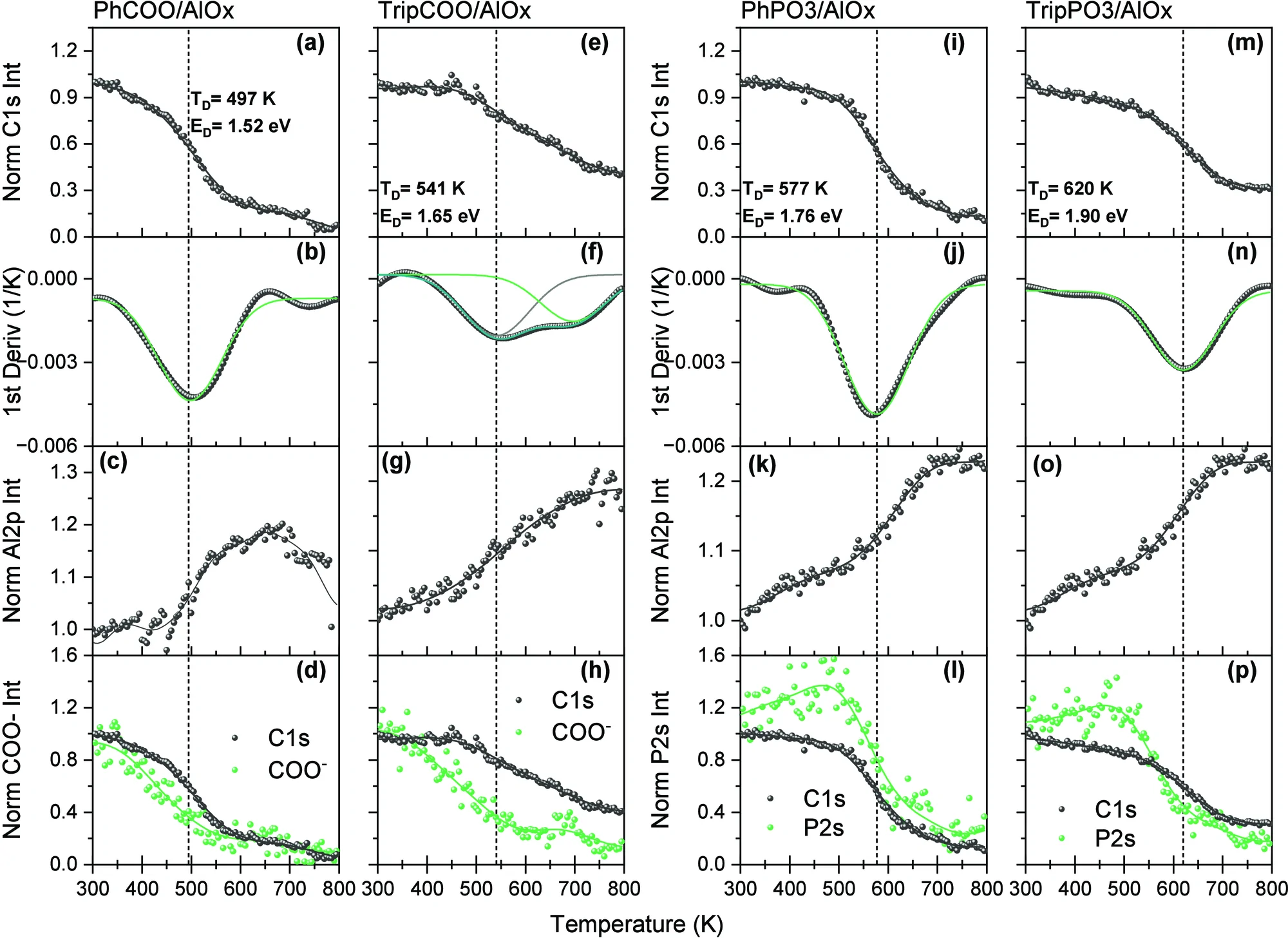
Example of TP-XPS data for PhCOO/AlO_*x*_ (a–d), TripCOO/AlO_*x*_ (e–h), PhPO_3_/AlO_*x*_ (i–l), and TripPO_3_/AlO_*x*_ (m–p). (a, e, i, m) Evolution of the C 1s intensity associated with the molecular backbones as a function of temperature. (b, f, j, n) Smoothed derivatives of the C 1s curves, used for *T*_D_ determination by fitting Gaussian peaks to the main minima. (c, g, k, o) Evolution of the Al 2p signal associated with the substrate as a function of temperature. (d, h) Evolution of the C 1s signals from molecular backbones (black circles) and CA anchoring group (green circles) as functions of temperature. (l, p) Evolution of the C 1s signal associated with the molecular backbones (black) and the P 2s signal associated with the PA anchoring groups (green) as functions of temperature. The vertical dashed lines indicate the *T*_D_ values, defined by the minima in (b), (f), (j), and (n).

The desorption-induced decrease of the main C 1s signal was accompanied by the respective increase in the Al 2p signal (Figure 3c), because of the reduced attenuation of this signal by the progressively thinning monolayer. The desorption process is also visible by the decrease of the minor C 1s component at ∼289.8 eV, related to the CA anchoring group (Figure 3d). We note that the respective signal drops faster with temperature than the component related to the phenyl moiety, which indicates that modification of the bonding configuration, causing the broadening and decrease in intensity of this weak signal, occurs already at temperatures below the desorption temperature. This effect is even more pronounced for TripCOO/AlO_*x*_ (Figure 3h). However, in this case, thermal analysis of the main C 1s signal shows a two-step desorption process with the first and second steps corresponding to ∼541 and ∼700 K, respectively (see data in Figures 3e,f). Since the desorption temperature depends on the heating rate and is, therefore, not strictly defined, we calculated the corresponding desorption energies (*E*_D_) using the Redhead equation^39^ (see the Supporting Information for details) to enable comparison of our results with available literature data (see Table 1). For PhCOO/AlO_*x*_ and TripCOO/AlO_*x*_, the calculated average desorption energies of 1.53 ± 0.09 and 1.68 ± 0.04 (first step), respectively, are both significantly higher than the analogous values for PhCOO/Ag (*E*_D_ ∼1.16 eV) and TripCOO/Ag (*E*_D_ ∼1.51 eV), estimated by TP-XPS as well.^18^ The enhancement of thermal stability induced by changing the substrate Ag→AlO_*x*_ is particularly pronounced for the PhCOOH SAMs. As the same experimental system and heating procedure were used in both the present study and ref 18, the difference in *E*_D_ directly translates into an increase in desorption temperature from 385 to 505 K, corresponding to an ∼120 K rise. For TripCOOH, the increase in thermal stability induced by the substrate change is smaller, but also clearly visible, amounting to ∼60 K. It is then particularly important that for the tripodal TripCOOH system, unlike for the monopodal PhCOOH, one can obtain a single-phenyl-ring-thin aromatic monolayer with high thermal stability on both metal (Ag) and insulating (AlO_*x*_) substrates using the same molecules. Considering well-documented possibilities of triptycene framework functionalization,^19^ such an option is highly relevant for OFET devices, as it could potentially enable both tuning the work function of the source/drain metal electrodes and the passivation of the gate electrode in a single-step SAM functionalization process. Such a possibility was demonstrated only very recently for OFETs with a self-aligned gate transistor structure using thicker aromatic monolayers with PA anchoring groups adsorbed on AlO_*x*_ (gate) and gold (source/drain) electrodes, with, however, unknown thermal stability.^1^ The analogous thermal stability analysis conducted for PhPO_3_/AlO_*x*_ (Figures 3i–l) and TripPO_3_/AlO_*x*_ (Figures 3m–p) demonstrates that the change from CA to PA leads to an increase in the desorption temperatures for both types of monolayers by ∼70 and ∼50 K, respectively (see the average values summarized in Table 1). Consequently, the corresponding average desorption energies reach values of 1.75 ± 0.02 (PhPO_3_/AlO_*x*_) and 1.86 ± 0.07 eV (TripPO_3_/AlO_*x*_). These values are very high compared with other types of SAMs analyzed with respect to this parameter, including systems with different anchoring groups (−S, −Se, −COO, −PO_3_, −C) and substrates (Au, Ag, AlO_*x*_).^18,28,40–48^ Furthermore, in the PhPO_3_/AlO_*x*_ and TripPO_3_/AlO_*x*_ cases, the tripodal configuration turns out to be thermally more stable than the monopodal one; however the difference between these two bonding configurations is slightly lower as for the CA-anchored SAMs. Interestingly, for both PhPO_3_/AlO_*x*_ and TripPO_3_/AlO_*x*_, the decrease in the P 2s signal originating from the anchoring groups correlates with the decrease in the C 1s signal (see Figure 3l and Figure 3p), indicating that the complete molecules desorb during the temperature ramping. As an additional reference for the measurements discussed above, we conducted a quantitative thermal stability analysis of aliphatic CA- and PA-anchored SAMs on AlO_*x*_, namely, CH_3_-(CH_2_)_15_-COO/AlO_*x*_ (HDCOO/AlO_*x*_) and CH_3_-(CH_2_)_15_-PO_3_/AlO_*x*_ (HDPO_3_/AlO_*x*_). Both these SAMs were studied and optimized by us earlier, and a preliminary study of their thermal stability was performed as well,^15,16^ even though with a less advanced XPS setup,^16^ which did not allow continuous heating and *in situ* spectral acquisition. Consequently, those experiments could not provide quantitative analysis enabling calculation of the desorption energy. In the present work, we address this limitation by performing a detailed TP-XPS characterization of these benchmark systems (see Figure S3), fully analogous to the measurements for the SAMs of the present study (Figure 3). The TP-XPS data for HDCOO/AlO_*x*_ reveal a two-step desorption process, with the first and second desorption steps corresponding to activation energies of 1.10 ± 0.03 and 1.50 ± 0.03 eV, respectively. These values, particularly that associated with the first desorption step, are much lower compared to the desorption energy for HDPO_3_/AlO_x_, which was determined from a well-defined single-step process and estimated at 1.79 ± 0.03 eV. Note that the corresponding desorption temperature of 585 ± 10 K is lower than the values previously reported for analogous PA-anchored aliphatic SAMs on Al_2_O_3_ (∼673 and ∼800 K, depending on the molecular backbone length).^49^ However, these studies cannot be directly compared with the present work because they employed a different heating protocol, which did not allow for the determination of the desorption energy. Generally, the thermal stability experiments conducted here provide two important conclusions. First, regardless of whether the molecular backbone is aromatic or aliphatic, the PA anchoring group affords substantially better thermal stability on AlO_*x*_ than the CA counterpart. Second, the ultrathin aromatic SAMs on AlO_*x*_ based on the triptycene framework and CA or PA anchoring group exhibit significantly higher thermal stability than the approximately twice-as-thick aliphatic SAMs employing the same anchoring chemistry; this effect is particularly large for the CA anchoring group (∼0.6 eV difference in *E*_D_).

To assess the chemical stability of TripCOO­(PO_3_)/AlO_*x*_ and PhCOO­(PO_3_)/AlO_*x*_, we investigated their susceptibility to exchange by aliphatic analogues bearing CA and PA anchoring groups, namely, HDCOO and HDPO_3_, respectively. The exchange process was monitored by consecutive XPS measurements of the monolayer thickness as a function of incubation time of a given monolayer (host) in a solution containing the guest molecules (HDCOO and HDPO_3_). In the case of exchange between the PA- and CA-anchored molecules, the intensity of the phosphorus signal (P 2s) was additionally monitored. Before analyzing the target ultrathin aromatic SAMs, we first evaluated the relative chemical stability of HDCOO/AlO_*x*_ and HDPO_3_/AlO_*x*_ through cross-exchange experiments. Figure 4a presents the evolution of the monolayer thickness and normalized P 2s intensity as functions of incubation time for the HDPO_3_/AlO_*x*_ monolayer exposed to a 1 mM solution of HDCOOH in isopropanol. Both parameters remain essentially constant, indicating a high resistance of HDPO_3_/AlO_*x*_ toward exchange by its CA counterpart. The results of the reverse experiment are shown in Figure 4b, illustrating the stability of the HDCOO/AlO_*x*_ monolayer against exchange by HDPO_3_ (1 mM HDPO_3_H_2_ solution in isopropanol). Although the apparent film thickness remains nearly unchanged with time, owing to the similar thicknesses of both monolayers, the normalized phosphorus signal gradually increases and eventually reaches saturation, clearly indicating progressive exchange. Since the phosphorus signal at saturation reaches approximately 90% of that observed for a fully formed HDPO_3_/AlO_*x*_ monolayer, the conversion of HDCOO/AlO_*x*_ into HDPO_3_/AlO_*x*_ can be considered nearly complete. Assuming that the phosphorus signal is proportional to the coverage of the AlO_*x*_ substrate by HDPO_3_ (0 ≤ *θ*_HDPO__3_ ≤ 1), the formation of an HDPO_3_/AlO_*x*_ monolayer can be described by using first-order Langmuir adsorption kinetics in the following form^18,50^

1 $$\theta_{HDPA}\left(t\right)=\theta_{SAT}\left(1-e^{-ckt}\right)$$

where *θ*_SAT_, *c*, and *k* are the saturation coverage, concentration of adsorbing molecules in solution (in mol L^–1^), and the adsorption rate (in L mol^–1^ s^–1^), respectively. For a given *c* of 1 mM, we obtained a rate constant of *k* ≈ 0.2. These experiments demonstrate a pronounced difference in the relative stability of CA- and PA-anchored aliphatic SAMs. While HDCOO/AlO_*x*_ is highly susceptible to exchange, HDPO_3_/AlO_*x*_ exhibits essentially complete exchange resistance under the applied conditions. The results of the exchange experiments for PhCOO­(PO_3_)/AlO_*x*_ and TripCOO­(PO_3_)/AlO_*x*_ in 1 mM HDPO_3_H_2_ solution in isopropanol are summarized in Figure 4c. As all investigated aromatic SAMs are significantly thinner than HDPO_3_/AlO_*x*_, the exchange process can be conveniently monitored following the increase in film thickness as a function of incubation time. The data reveal the largest thickness increase for PhCOO/AlO_*x*_, reaching a final value of ∼14.2 Å. Within the uncertainty of the thickness determination (±0.5 Å), this value matches 70–80% of the HDPO_3_/AlO_*x*_ thickness, indicating a significant extent of the molecular exchange. Replacing the phenyl backbone with the triptycene framework while retaining the CA anchoring group (TripCOO/AlO_*x*_) results in a lower final thickness of ∼13.2 Å, suggesting reduced exchange efficiency, i.e., better chemical stability. In contrast, the use of PA instead of CA as the anchoring group significantly enhances monolayer stability of both phenyl- and triptycene-based systems. At the maximum incubation time, the film thicknesses reached only ∼12.0 and ∼10.5 Å for PhPO_3_/AlO_*x*_ and TripPO_3_/AlO_*x*_, respectively (Figure 4c). The lower value for the latter system underlines, once more, a positive effect of the tripodal anchoring on SAM stability. Replacing the exchange solution from HDPO_3_H_2_ to 1 mM HDCOOH in isopropanol yields the same general stability trends, although the differences become considerably more pronounced (Figure 4d). For PhCOO/AlO_*x*_, a significant exchange by HDCOO can again be inferred from the final thickness value (∼14.3 Å). In contrast, the exchange process is essentially suppressed for TripCOO/AlO_*x*_, as the film thickness remains essentially unchanged within the experimental uncertainty, which is not better than 10–15% considering the multiple washing and incubation of the same samples. Similarly, the use of PA instead of CA as the SAM anchoring group leads to only a limited extent of exchange in the PhPO_3_/AlO_*x*_ case, reflected by an increase in thickness to ∼10.1 Å, while no significant thickness change, and thus no detectable exchange, is observed for TripPO_3_/AlO_*x*_.

**Figure 4:**
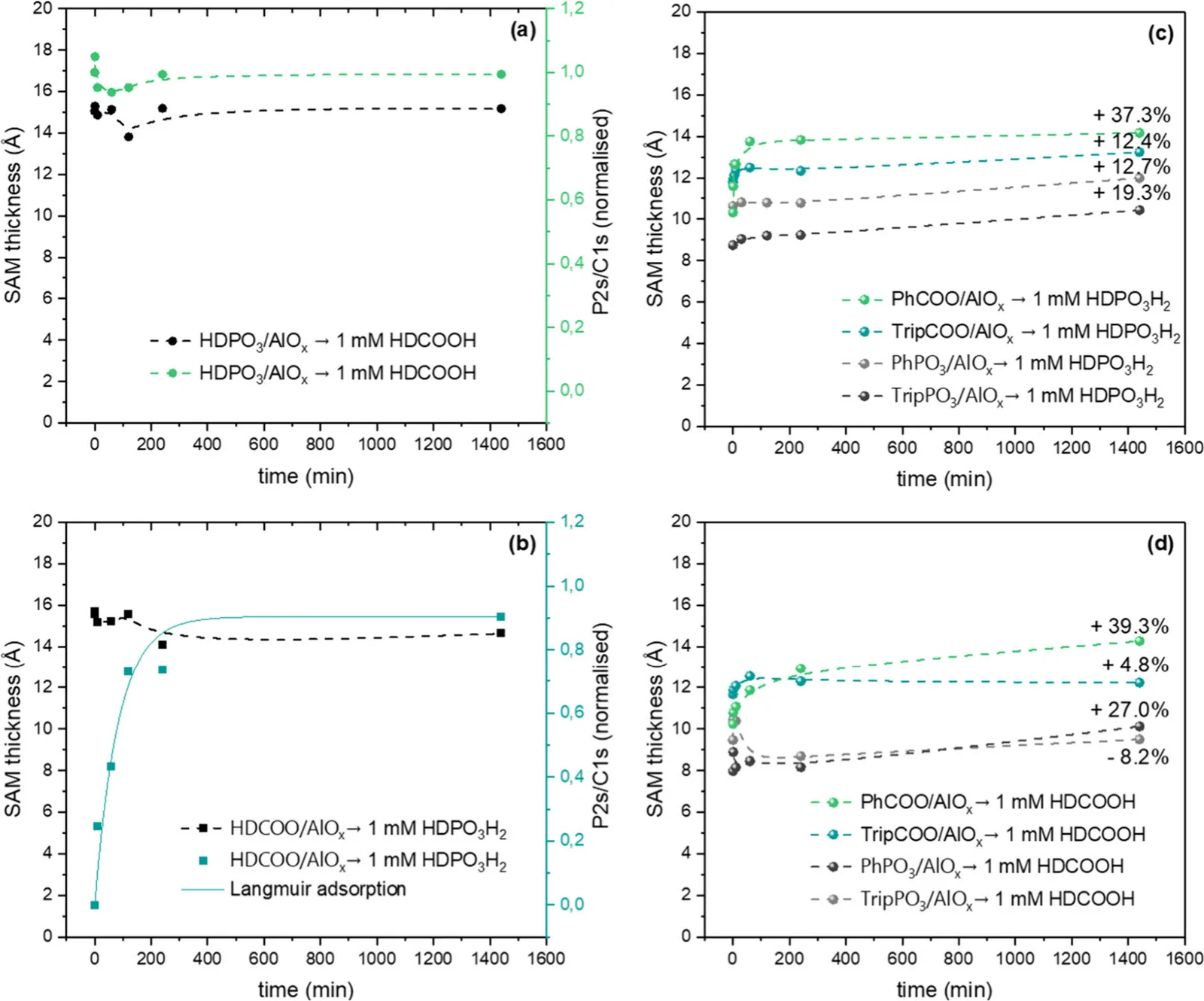
Summary of chemical stability data. The monolayer thickness and phosphorus (P 2s) signal are presented as functions of incubation time for the primary SAMs incubated in 1 mM HDCOOH or HDPO_3_H_2_ solutions. The legends are given in the plots. (a) HDPO_3_/AlO_*x*_ in HDCOOH solution; (b) HDCOO/AlO_*x*_ in HDPO_3_H_2_ solution; (c) TripCOO­(PO_3_)/AlO_*x*_ and PhCOO­(PO_3_)/AlO_*x*_ in HDPO_3_H_2_ solution; and (d) TripCOO­(PO_3_)/AlO_*x*_ and PhCOO­(PO_3_)/AlO_*x*_ in HDCOOH solution.

In summary, XPS and NEXAFS spectroscopy confirmed the successful formation of well-defined, upright-oriented monolayers with phenyl and triptycene backbones and CA and PA anchoring groups on the surface of naturally oxidized Al. The AlO_*x*_ overlayer was ∼1 nm thick and had a stoichiometry close to Al_2_O_3_. Importantly, all the studied SAMs exhibited similar thickness (∼1 nm) and surface density of upright oriented aromatic units. These results enabled a meaningful comparison of the relative stability of these ultrathin aromatic monolayers deposited on a ∼1 nm-thick dielectric layer, particularly addressing the impact of the anchoring group type (CA vs PA) and bonding mode (monodentate vs tridentate) on the thermal and chemical stability of these monolayers. The dedicated TP-XPS experiments demonstrate significantly higher desorption energies (by approximately 0.2 eV) for the PA-anchored monolayers with phenyl and triptycene backbones compared to the CA-anchored ones, independent of the bonding mode. This effect was even more pronounced for long aliphatic SAMs on the same substrate (∼0.7 eV for PA vs CA), confirming its general character. On the other hand, the TP-XPS data suggest that the tripodal bonding configuration increases the desorption energy by ∼0.15 eV in the CA case and by ∼0.11 eV in the PA case compared to the monopodal case, so the effect is similar for both applied anchoring groups.

The crucial roles of both the anchoring group and bonding mode are evident in the chemical stability of the SAMs, tested by the exchange experiments. The PA-anchored SAMs exhibit much higher stability against molecular exchange compared to their CA-anchored counterparts, with the tripodal bonding configuration contributing positively to the stability in both the CA and PA cases. The most stable monolayer, TripPO_3_/AlO_*x*_, exhibits no or only a minimal exchange in 1 mM solutions of HDCOOH and HDPO_3_H_2_, respectively.

This establishes a clear strategy for optimizing the thermal and chemical stability of ultrathin aromatic films on oxide application-relevant substrates, taking naturally oxidized aluminum with an ∼1 nm thick AlO_*x*_ overlayer as a representative case. This strategy relies on a combination of PA anchoring and a tripodal bonding configuration, resulting in a record-high desorption energy (∼1.9 eV) for the test TripPO_3_/AlO_*x*_ system, comparable to the most stable carbene-based SAMs on Au substrates,^42^ while also offering high resistance to molecular exchange in solutions. Consequently, TripPO_3_ SAMs, decorated optionally with suitable functional tail groups, are well-suited for gate electrode engineering in OTFTs and, likely, other oxide electrodes and interlayers in electronic and photovoltaic devices. In contrast to the PA-anchored SAMs, CA-anchored ones turned out to be less stable, but, due to their applicability to both oxide and metal substrates, suitable for one-step engineering of both conductive metal electrodes like silver and ultrathin dielectric coatings such as AlO_*x*_, as in self-aligned gate transistor structures.^1^ The TripCOO monolayers provide an attractive solution to this purpose. They offer ultrathin aromatic platforms with reasonably high chemical stability and notably high desorption energies of approximately 1.51 eV (TripCOO/Ag)^18^ and 1.65 eV (TripCOO/AlO_*x*_), respectively.

## Supplementary Material



## Notes

Sample preparation procedure, XPS/TP-XPS/NEXAFS experimental details, SAMs and AlO_x_ analysis of thickness and stoichiometry by XPS, the average molecular tilt angle estimation by NEXAFS spectroscopy, the desorption energy estimation by TP-XPS, TP-XPS of the reference samples (PDF)
