# Supplementary material for: Ultrathin and Stable Aromatic Monolayers on Naturally Oxidized Aluminum: The Role of Type and Number of Anchoring Groups
Source: J Phys Chem Lett. 2026 Sep 16;17(39):11261–70. doi: 10.1021/acs.jpclett.6c02547 (PMC13637071; doi:10.1021/acs.jpclett.6c02547)
Supplement: Supplementary file 1 [file jz6c02547_si_001.pdf]

Supporting Information

Ultrathin and Stable Aromatic Monolayers on Naturally Oxidized Aluminum – The Role of Type and Number of Anchoring Groups.

Anna Rojek,[§] *Marta Szatny,*[§] *Daria M. Cegiełka,*[§] *Łukasz Bodek,*[§] *Yoshiaki Shoji,*[#,‡] *Takanori Fukushima*[#,‡]* *Michael Zharnikov,*[†]* and *Piotr Cyganik*[§]*

[§] Jagiellonian University, Faculty of Physics, Astronomy and Applied Computer Science, Smoluchowski Institute of Physics, Łojasiewicza 11, 30-348 Kraków, Poland

[#] Laboratory for Chemistry and Life Science, Institute of Integrated Research, Institute of Science Tokyo, Yokohama 226-8501, Japan

[‡] Research Center for Autonomous Systems Materialogy (ASMat), Institute of Integrated Research, Institute of Science Tokyo, Yokohama 226-8501, Japan

[†] Angewandte Physikalische Chemie, Universität Heidelberg, Im Neuenheimer Feld 253, D-69120 Heidelberg, Germany

1. Sample preparation

Two types of naturally oxidized aluminum substrates were prepared by evaporation of Al (purity 99.999%, Kurt J. Lesker) film on either silicon wafer (Si-Mat, Germany) or mica (V1 grade, Ted Pella, USA). The AlO_x/mica substrates were used for the thermal stability studies. For both types of substrates, ~100 nm of Al film (rate ~1.2–1.6 nm/s) was evaporated at room temperature (RT) on silicon and at ~360°C on mica. The silicon wafers and mica substrates were pre-annealed for ~24 hours before evaporating aluminum (to remove water and surface contamination) at ~100 and ~360 °C, respectively. The gold substrates on Si wafers, for the reference HDT/Au samples, were prepared by evaporation of ~100 nm Au film (RT, rate ~0.1 nm/s) with ~5 nm adhesive Cr layer (RT, rate ~0.01 nm/s). It is worth noting that the freshly prepared Au substrates were processed in air; meanwhile, to minimize the impact of the oxide and humidity from the atmosphere, aluminum substrates were directly transferred from the evaporation chamber to a glovebox (MBraun) in an argon atmosphere.

The 1,8,13-tricarboxytriptycene (TripCOOH) and 1,8,13-tris(phosphonic-methyl)triptycene ($TripPO_3H_2$) were synthesized using the previously reported procedures.[1] Benzenoic acid (PhCOOH, >99.5%) and benzyl phosphonic acid ($PhPO_3H_2$, >97%) were purchased from Sigma-Aldrich and used as received.

For SAMs formation, freshly prepared AlO_x substrates were incubated for 24 hours at RT in the isopropyl alcohol (Eurochem BDG, Poland, p.a. grade) solution of TripCOOH (0.1 mM), $TripPO_3H_2$ (0.1 mM), PhCOOH (1.0 mM), or $PhPO_3H_2$ (1.0 mM) and subsequently washed with isopropyl alcohol, followed by drying in the nitrogen stream. Additionally, as the reference samples for the thermal stability and exchange experiments, $HDPO_3/AlO_x$ and $HDCOO/AlO_x$ monolayers were obtained by immersion (24 h at RT) of Al substrates into isopropyl alcohol solution (1.0 mM) of hexadecylphosphonic acid (>97%, Sigma-Aldrich) and heptadecanoic acid (>99%, Sigma-Aldrich), respectively.[2,3] As a reference for the XPS experiments, 1-hexadecanethiol (HDT, 97% Alfa Aesar) SAMs on Au were prepared by 24 h incubation of the Au substrates in 1.0 mM solution of HDT at RT.

2. XPS analysis

The experiments were carried out using a ThermoFisher Scientific ESCALAB™ QXi Microprobe workstation equipped with Al Kα X-ray source (E = 1486.6 eV, spot size = 650 µm). The measurements were performed under ultrahigh vacuum (a base pressure of < 5 × 10^{-10} mbar) and in normal emission geometry with a 0.1 eV energy step. The Au $4f_{7/2}$ peak at 84.0 eV was used to calibrate the binding energy scale, and its full width at half maximum (FWHM) was employed to estimate the energy resolution, *i.e.* ~0.5 eV after the correction for the natural FWHM of the Au $4f_{7/2}$ peak (0.42 eV).[4] The spectra were acquired and processed using the Thermo Scientific Avantage® software. Individual peaks were fitted with pseudo-Voigt profiles.

For the thermal stability test by TP-XPS, the previously established procedure was applied.[5] In short, the samples were heated at a heating rate of ~3.6 K/min, and the XPS measurements were conducted at every 5 K step. To minimize the temperature variation during the spectra acquisition, the time for a single measurement (substrate signal, carbon signal) was set to only ~27 seconds, corresponding to the temperature variation by 1.7 K. Individual spectra were collected at different sample spots taken from the matrix of points evenly distributed over an area of ~6 mm^2 (to limit the potential irradiation-induced damage at elevated temperatures).[6] To verify the sample homogeneity within this area, the substrate signal in the points of the matrix used for the thermal analysis was measured at RT before the TP-XPS analysis, indicating excellent sample homogeneity. For each SAM type, the TP-XPS analysis was conducted for 3-5 different samples, which enabled calculation of an average value and the related error for the desorption temperature (and the derived desorption energy), defined as the maximal difference between the average and measured values.

2.1 SAMs thickness calculation. The thickness of the self-assembled monolayers (SAMs) on naturally oxidized aluminum (AlO_x/Al) was determined by the comparison of C 1s signal with a reference

monolayer of 1-hexadecanethiol on gold (HDT/Au) as the well-known system.[2,3] For this purpose, the following equation was used (S1):

$$\frac{I_{C1s}^{SAM/AlO_x}}{I_{C1s}^{HDT/Au}} = \frac{1-\exp\left(-\frac{(d_{SAM/AlO_x}-z_{SAM/AlO_x})}{\lambda_{C1s}}\right)}{1-\exp\left(-\frac{(d_{HDT/Au}-z_{HDT/Au})}{\lambda_{C1s}}\right)} \text{ (S1)}$$

$$\lambda_{C1s} = 0.3\, E_{kC1s}^{0.64} \text{ (S2)}$$

$$E_{kC1s} = h\nu - \Phi_{XPS} - E_{bC1s} \text{ (S3)}$$

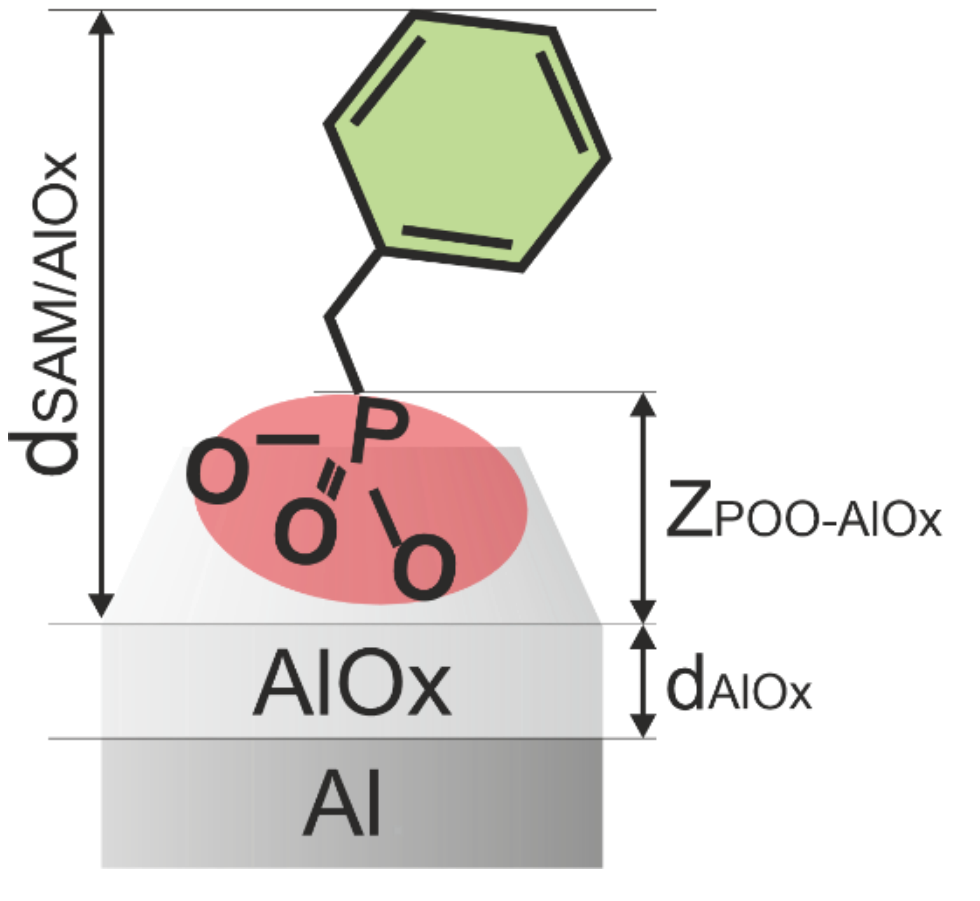

Figure S1. Schematic representation of SAM thickness calculation using $PhPO_3/AlO_x$ as an example.

Where I_{C1s}^{SAM/AlO_x} and $I_{C1s}^{HDT/Au}$ are the total intensities of the C 1s signals of the SAM/AlO_x and reference HDT/Au, respectively, z_i is the geometric length of the bonding group [Å] (the distance between the substrate and the first carbon of SAM backbone, **Figure S1**), d_{SAM/AlO_x} is the SAM/AlO_x thickness [Å], $d_{HDT/Au}$ is the HDT/Au monolayer thickness (~19.7 Å [7]), λ_{C1s} is the average electron attenuation length for the photoelectrons emitted from C 1s [Å] (~28 Å at this kinetic energy [8]), E_{kC1s} is the kinetic energy of the C1s electrons [eV], $h\nu$ is the photon energy [eV] (~1486.6 eV for Al Kα), Φ_{XPS} is the work function of the spectrometer [eV] (~4.92 eV), and E_{bC1s} is the binding energy of the C1s signal. The error of thickness was estimated using few independent measurements by ~5% of measured value.

2.2 Aluminum oxide layer thickness calculation. The thickness of the aluminum oxide layer which was naturally formed on the metallic aluminum (d_{AlO_x} [Å]) was calculated using the following formula (S4).[2,3]

$$\frac{I_{Al2p}^{oxide}}{I_{Al2p}^{metal}} = K \times \frac{1-exp\left(-\frac{d_{AlO_x}}{\lambda_{Al2O3}}\right)}{exp\left(-\frac{d_{AlO_x}}{\lambda_{Al2O3}}\right)} \quad \text{(S4)}$$

where I_{Al2p}^{oxide} and I_{Al2p}^{metal} are the intensities of the peaks corresponding to the oxidized (~75.5 eV) and metallic (~72.8 eV) aluminum components, respectively, λ_{Al2O3} is the average electron mean free path through the Al_2O_3 (~25 Å[9]), and K is an experimental constant obtained using the AlO_x film with known thickness [10].

2.3 Aluminum oxide stoichiometry. The stoichiometry of aluminum oxide layer (O/Al ratio) was calculated using the following equation considering signal attenuation (S5) [11]:

$$\frac{O}{Al} = \frac{\frac{I_{O1s}}{SF_{O1s} \times T_{O1s}}}{\frac{I_{Al2p}^{oxide}}{SF_{Al2p} \times T_{Al2p}}} \frac{\left[1-exp\left(-\frac{d_{oxide}}{\lambda_{Al2O3}^{Al2p}}\right)\right]\left[exp\left(-\frac{d_{SAM}}{\lambda_{SAM}^{Al2p}}\right)\right]}{\left[1-exp\left(-\frac{d_{oxide}}{\lambda_{Al2O3}^{O1s}}\right)\right]\left[exp\left(-\frac{d_{SAM}}{\lambda_{SAM}^{O1s}}\right)\right]} \quad \text{(S5)}$$

where I_{O1s} and I_{Al2p}^{oxide} are the intensities of the O 1s and the Al 2p oxide components, respectively, corrected using the corresponding Scofield factor (SF_i), and the transmission function (T_i) obtained from the Avantage® software. The thickness of the AlO_x layer (d_{oxide}) and SAM (d_{SAM}) were determined as the described in the previous sections, and the corresponding electron inelastic mean free paths for Al 2p and O 1s photoelectrons in Al_2O_3 [9] and SAM [8] (λ_{Al2O3}^{Al2p}, λ_{Al2O3}^{O1s}, λ_{SAM}^{Al2p}, and λ_{SAM}^{O1s}, respectively).

2.4 Desorption energy estimation by TP-XPS. To compare the thermal desorption energies of the SAMs, the intensity of the main component of the C 1s spectra was normalized to its initial value at room temperature and plotted as a function of temperature. The resulting curves were smoothed using an FFT filter and subsequently analyzed by calculating the first derivative. The desorption temperature, T_D, and its associated uncertainty (standard deviation) were determined by fitting a Gaussian function to the

minimum of the derivative curves. Finally, the desorption energy, E_D [eV], and the corresponding error were calculated using the Redhead equation (S6) [12]:

$$E_D = k_B T_D \left[\ln\left(\frac{v_T\, T_D}{h_r}\right) - 3.64\right] \text{(S6)}$$

Where k_B is the Boltzmann constant, v_T pre-exponential (frequency) factor, typically taken as 10^{13} Hz for SAMs [1,13–19] and h_r is the heating rate (~3.6 K/min). The absolute desorption energies depend on the assumed pre-exponential factor and therefore carry a corresponding systematic uncertainty, whereas the relative comparison among the SAMs investigated here is hardly affected by the choice of v_T because identical kinetic parameters were used throughout the data analysis for all types of SAMs studied (see discussion in ref. [20]

3. NEXAFs analysis

The NEXAFS spectra at the C K-edge were recorded at the HE-SGM beamline of the BESSY II synchrotron radiation facility using linearly polarized light (~90% polarization). The measurements were performed in partial electron yield mode with a retarding voltage of –150 V and an energy resolution of ~0.3 eV. To determine the molecular orientation, the incidence angle of the X-ray beam was varied from 90° to 20° relative to the surface plane. Spectra were normalized against a freshly sputtered indium tin oxide reference to correct for photon flux variations. Further processing included setting the pre-edge baseline to zero and normalizing the post-edge region to unity. The photon energy scale was calibrated using the π* resonance of highly oriented pyrolytic graphite (HOPG) at 285.38 eV.[21]

3.1 Tilt angle estimation

PhCOO/AlO$_x$ and PhPO$_3$/AlO$_x$

For a vector-like transition orbital on a substrate exhibiting threefold (or higher) symmetry, the angular dependence of the absorption resonance intensity on the X-ray incidence angle (θ) can be described by equation (S7)[22]:

$$I(\theta) = A\left\{P\frac{1}{3}\left[1 + \frac{1}{2}(3\cos^2\theta - 1)(3\cos^2\alpha - 1)\right] + (1-P)\frac{1}{2}\sin^2\alpha\right\} \quad (S7)$$

where A is an orbital-dependent proportionality constant, P is the degree of X-ray polarization (0.9), and α is the angle between the vector orbital (π^*_{Ph}) and the surface normal. This angle can be further converted into the average molecular tilt angle (β) by assuming a defined molecular twist angle (σ) (**Figure 2** and **Figure S2**).

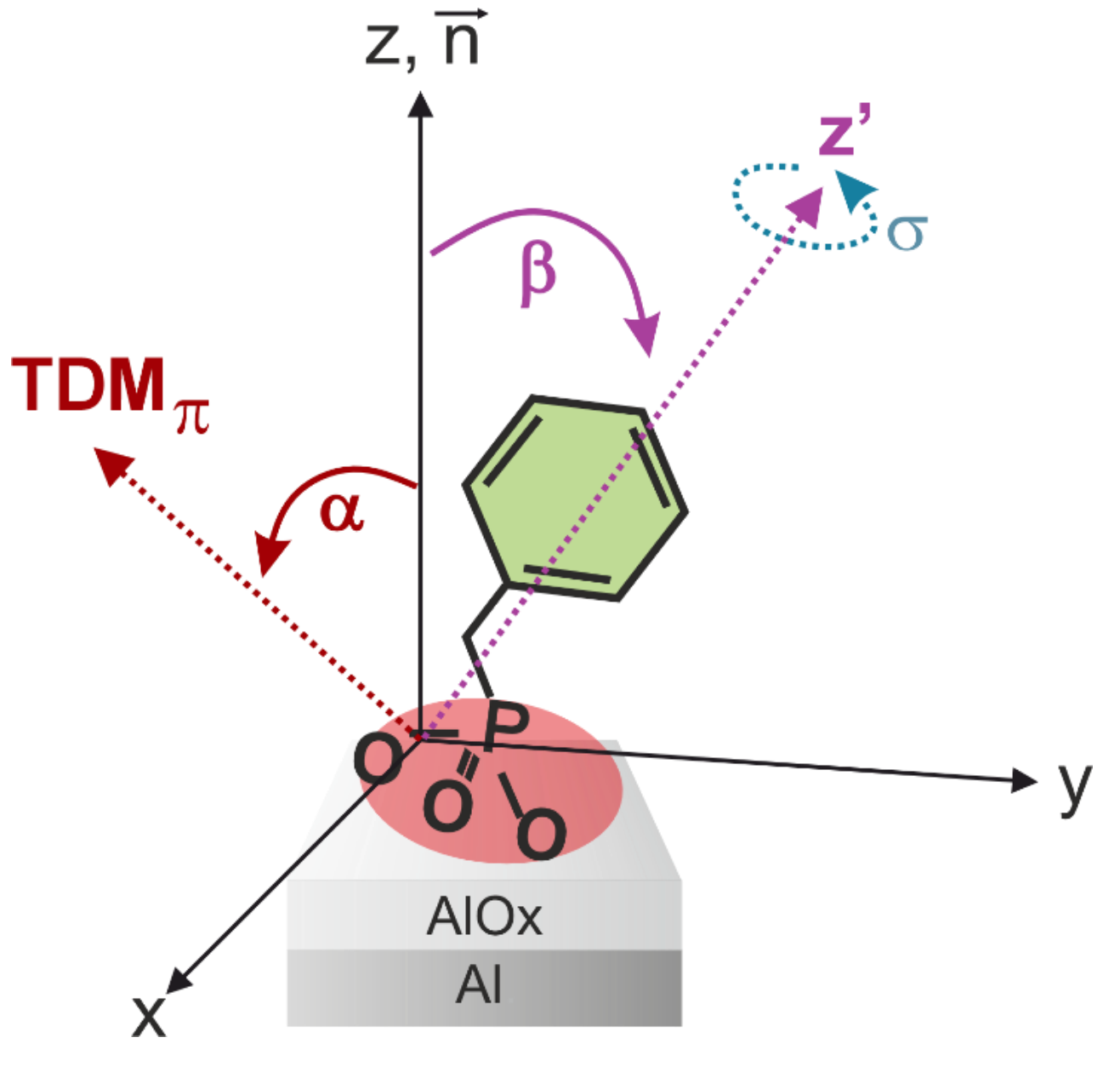

Figure S2. Schematic representation of the relevant angles used for the analysis of molecular orientation in representative SAM ($PhPO_3/AlO_x$), where the z-axis corresponds to the surface normal of the metal substrate. The main molecular axis (z') is tilted with respect to the surface normal (z-axis) by the angle β, while the transition dipole moment (TDM_π) vector is tilted by the angle α. Rotation of the molecule about its main axis is described by the twist angle σ, which is defined as zero when the tilt plane is perpendicular to the plane of the phenyl ring.

Because the absolute value of A is not known, relative intensities were evaluated, i.e., I(θ)/I(20°), I(θ)/I(30°), I(θ)/I(55°), and I(θ)/I(90°), for the π^*_{Ph} resonance. These ratios were fitted to extract the corresponding α values. The average of the α values obtained from the different angular ratios was then used to calculate the mean β using following equation:

$$\sin(\beta) = \frac{\cos(\alpha)}{\cos(\sigma)} \qquad (S8)$$

where $\sigma = 32°$ is the twist angle characteristic of bulk phenyl [23].

The error of tilt angle calculation using few independent measurements was estimated by ± 3°.

TripCOO/AlO_x and $TripPO_3/AlO_x$

For triptycene-based SAMs, the intrinsic threefold symmetry of the molecule allows a more straightforward analysis. Each of the three aromatic rings contributes an independent NEXAFS signal of identical intensity, with the corresponding transition dipole moments rotated by 120° relative to one another (**Figure 2**). The total resonance intensity can therefore be expressed by the equation (S9) [24]:

$$I(\theta) = \frac{3B}{2} P(3\sin^2\beta - 1)\cos^2\theta + \frac{3B}{2}\left(1 - \frac{1}{2}\sin^2\beta\right) \text{(S9)}$$

where B is a scaling factor and P represents the X-ray polarization factor. Plotting the normalized resonance intensity as a function of $\cos^2\theta$ yields a linear relationship, enabling direct extraction of the molecular tilt angle β using the equation (S10) [24]:

$$\beta = \sin^{-1}\left(\sqrt{\frac{2m+2aP}{m+3aP}}\right) \text{(S10)}$$

with m and a are the slope and intercept of the linear fit, respectively. This approach allows direct determination of β without requiring an assumed value of σ. The final molecular tilt angle was obtained by averaging the β values derived from fits of the I(θ)/I(20°), I(θ)/I(30°), I(θ)/I(55°), and I(θ)/I(90°) intensity ratios. The error of tilt angle calculation using few independent measurements was estimated by ± 3°.

4. Thermal desorption for the reference samples

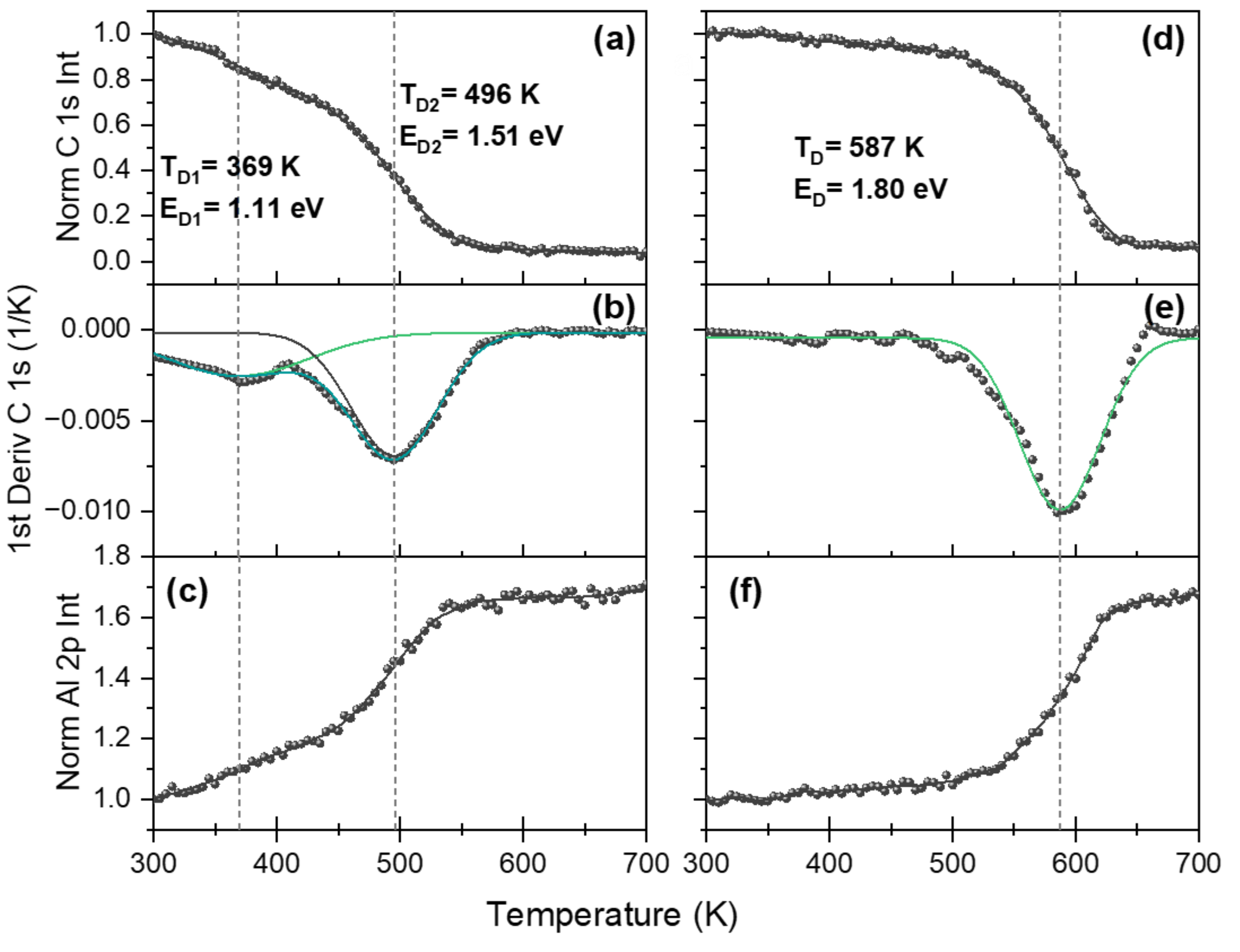

Figure S3. Representative TP-XPS data for HDCOO/AlO_x and $HDPO_3/AlO_x$ (a-f), HDCOO/AlOx (e-c), $HDPO_3/AlO_x$ (d-f): temperature evolution of the C 1s intensity associated with the aliphatic chain (a) and (d); smoothed derivatives of the C 1s curves, used for TD determination by fitting Gaussian peaks to the main minima; (b) and (e); temperature evolution of the Al 2p intensity associated with the substrate (c) and (f). The vertical dashed lines indicate the TD values, defined by the minima in (b) and (e).

References

[1] S. Das, G. Nascimbeni, R.O. De La Morena, F. Ishiwari, Y. Shoji, T. Fukushima, M. Buck, E. Zojer, M. Zharnikov, Porous Honeycomb Self-Assembled Monolayers: Tripodal Adsorption and Hidden Chirality of Carboxylate Anchored Triptycenes on Ag, ACS Nano 15 (2021) 11168–11179. https://doi.org/10.1021/acsnano.1c03626.

[2] D.M. Cegiełka, K. Kozieł, M. Zharnikov, P. Cyganik, Anchoring groups for ordered and highly stable monomolecular films on naturally oxidized aluminum – phosphonate versus carboxylate, Appl. Surf. Sci. (2024) 160199. https://doi.org/10.1016/j.apsusc.2024.160199.

[3] D.M. Cegiełka, K. Kozieł, M. Zharnikov, P. Cyganik, Molecular engineering of technologically relevant surfaces - carboxylic acids on naturally oxidized aluminum, Appl. Surf. Sci. 636 (2023) 157798. https://doi.org/10.1016/j.apsusc.2023.157798.

[4] K. Heister, M. Zharnikov, M. Grunze, L.S.O. Johansson, Adsorption of alkanethiols and biphenylthiols on Au and Ag substrates: A high-resolution X-ray photoelectron spectroscopy study, J. Phys. Chem. B 105 (2001) 4058–4061. https://doi.org/10.1021/jp010127q.

[5] D.M. Cegiełka, Ł. Bodek, M. Zharnikov, P. Cyganik, Odd-Even Effects in the Structure and Thermal Stability of Carboxylic Acid Anchored Monolayers on Naturally Oxidized Aluminum Surface, J. Phys. Chem. Lett. 16 (2025) 3343–3350. https://doi.org/10.1021/acs.jpclett.5c00500.

[6] M. Zharnikov, Importance of Electrostatic Effects for Interpretation of X-ray Photoemission Spectra of Self-Assembled Monolayers, Adv. Mater. Interfaces 12 (2025). https://doi.org/10.1002/admi.202400595.

[7] H.U. Müller, M. Zharnikov, B. Völkel, A. Schertel, P. Harder, M. Grunze, Low-Energy Electron-Induced Damage in Hexadecanethiolate Monolayers, J. Phys. Chem. B 102 (1998) 7949–7959. https://doi.org/10.1021/jp981886k.

[8] C.L.A. Lamont, J. Wilkes, Attenuation Length of Electrons in Self-Assembled Monolayers of *n* -Alkanethiols on Gold, Langmuir 15 (1999) 2037–2042. https://doi.org/10.1021/la981168p.

[9] M.A. Flores-Mancera, J.S. Villarrubia, G. Massillon-JL, Electron Inelastic Mean Free Paths for LiF, CaF_2, Al_2O_3, and Liquid Water from 433 keV down to the Energy Gap, ACS Omega 5 (2020) 4139–4147. https://doi.org/10.1021/acsomega.9b03872.

[10] E. Tan, P.G. Mather, A.C. Perrella, J.C. Read, R.A. Buhrman, Oxygen stoichiometry and instability in aluminum oxide tunnel barrier layers, Phys. Rev. B 71 (2005) 161401. https://doi.org/10.1103/PhysRevB.71.161401.

[11] D.M. Cegiełka, K. Kozieł, M. Zharnikov, P. Cyganik, Anchoring groups for ordered and highly stable monomolecular films on naturally oxidized aluminum – phosphonate versus carboxylate, Appl. Surf. Sci. 665 (2024) 160199. https://doi.org/10.1016/j.apsusc.2024.160199.

[12] P.A. Redhead, Thermal desorption of gases, Vacuum 12 (1962) 203–211. https://doi.org/10.1016/0042-207X(62)90978-8.

[13] C.M. Crudden, J.H. Horton, M.R. Narouz, Z. Li, C.A. Smith, K. Munro, C.J. Baddeley, C.R. Larrea, B. Drevniok, B. Thanabalasingam, A.B. McLean, O. V. Zenkina, I.I. Ebralidze, Z. She, H.-B. Kraatz, N.J. Mosey, L.N. Saunders, A. Yagi, Simple direct formation of self-assembled N-heterocyclic carbene monolayers on gold and their application in biosensing, Nat. Commun. 7 (2016) 12654. https://doi.org/10.1038/ncomms12654.

[14] C.R. Larrea, C.J. Baddeley, M.R. Narouz, N.J. Mosey, J.H. Horton, C.M. Crudden, *N* -Heterocyclic Carbene Self-assembled Monolayers on Copper and Gold: Dramatic Effect of Wingtip Groups on Binding, Orientation and Assembly, ChemPhysChem 18 (2017) 3536–3539. https://doi.org/10.1002/cphc.201701045.

[15] A. Krzykawska, M. Wróbel, K. Kozieł, P. Cyganik, N-Heterocyclic Carbenes for the Self-Assembly of Thin and Highly Insulating Monolayers with High Quality and Stability, ACS Nano 14 (2020) 6043–6057. https://doi.org/10.1021/acsnano.0c01733.

[16] M. Kruk, D.M. Cegiełka, M. Wróbel, M. Zharnikov, P. Cyganik, Ultra-Dense, Highly Ordered and Thermally Stable Anthracene Monolayers on Ag(111)─The Impact of the Anchoring Group, J. Phys. Chem. C 129 (2025) 4272–4281. https://doi.org/10.1021/acs.jpcc.4c08566.

[17] M. Wróbel, D.M. Cegiełka, A. Asyuda, K. Kozieł, M. Zharnikov, P. Cyganik, N-heterocyclic carbenes – The design concept for densely packed and thermally ultra-stable aromatic self-assembled monolayers, Nano Today 53 (2023) 102024. https://doi.org/10.1016/j.nantod.2023.102024.

[18] M. Wróbel, T. Żaba, E. Sauter, M. Krawiec, J. Sobczuk, A. Terfort, M. Zharnikov, P. Cyganik, Thermally Stable and Highly Conductive SAMs on Ag Substrate—The Impact of the Anchoring Group, Adv. Electron. Mater. 7 (2021). https://doi.org/10.1002/aelm.202000947.

[19] A. Rojek, D.M. Cegiełka, M. Wróbel, M. Stępień, Y. Shoji, T. Fukushima, M. Zharnikov, P. Cyganik, Formation of Ultrathin and Highly Stable Aromatic Monolayers on Silver Surface─Three Legs Are Better Than One, ACS Appl. Mater. Interfaces 18 (2026) 15665–15675. https://doi.org/10.1021/acsami.5c19530.

[20] P.E. Batson, Carbon 1s near-edge-absorption fine structure in graphite, Phys. Rev. B 48 (1993) 2608–2610. https://doi.org/https://doi.org/10.1103/PhysRevB.48.2608.

[21] J. Stohr, NEXAFS Spectroscopy, Springer Series in Surface Science, 2003.

[22] C. Zhang, S. Das, N. Sakurai, T. Imaizumi, S. Sanjayan, Y. Shoji, T. Fukushima, M. Zharnikov, Phosphonic acid anchored tripodal molecular films on indium tin oxide, Physical Chemistry Chemical Physics 26 (2024) 11360–11369. https://doi.org/10.1039/D4CP00892H.

[23] J. Liu, M. Kind, B. Schüpbach, D. Käfer, S. Winkler, W. Zhang, A. Terfort, C. Wöll, Triptycene-terminated thiolate and selenolate monolayers on Au(111), Beilstein Journal of Nanotechnology 8 (2017) 892–905. https://doi.org/10.3762/bjnano.8.91.
